# Characterization of Nanobody Binding to Distinct Regions of the SARS-CoV-2 Spike Protein by Flow Virometry

**DOI:** 10.3390/v17040571

**Published:** 2025-04-15

**Authors:** Mariam Maltseva, Martin A. Rossotti, Jamshid Tanha, Marc-André Langlois

**Affiliations:** 1 Department of Biochemistry, Microbiology and Immunology, Faculty of Medicine, University of Ottawa, Ottawa, ON K1H 8M5, Canada; mmalt049@uottawa.ca (M.M.); jamshid.tanha@nrc-cnrc.gc.ca (J.T.); 2 Human Health Therapeutics Research Centre, Life Sciences Division, National Research Council Canada, Ottawa, ON K1N 1J1, Canada; martin.rossotti@nrc-cnrc.gc.ca; 3 uOttawa Center for Infection, Immunity, and Inflammation (CI3), Ottawa, ON K1H 8L1, Canada

**Keywords:** flow virometry, nanobodies, diagnostics, antivirals, SARS-CoV-2

## Abstract

Nanobodies, or single-domain antibodies (V_H_Hs) from camelid heavy-chain-only antibodies, offer significant advantages in therapeutic and diagnostic applications due to their small size and ability to bind cryptic protein epitopes inaccessible to conventional antibodies. In this study, we examined nanobodies specific to regions of the SARS-CoV-2 spike glycoprotein, including the receptor-binding domain (RBD), N-terminal domain (NTD), and subunit 2 (S2). Using flow virometry, a high-throughput technique for viral quantification, we achieved the efficient detection of pseudotyped viruses expressing the spike glycoprotein. RBD-targeting nanobodies showed the most effective staining, followed by NTD-targeting ones, while S2-specific nanobodies exhibited limited resolution. The simple genetic structure of nanobodies enables the creation of multimeric formats, improving binding specificity and avidity. Bivalent V_H_H-Fc constructs (V_H_Hs fused to the Fc region of human IgG) outperformed monovalent formats in resolving viral particles from background noise. However, S2-specific monovalent V_H_Hs demonstrated improved staining efficiency, suggesting their smaller size better accesses restricted antigenic sites. Furthermore, direct staining of cell supernatants was possible without virus purification. This versatile nanobody platform, initially developed for antiviral therapy against SARS-CoV-2, can be readily adapted for flow virometry applications and other diagnostic assays.

## 1. Introduction

The impact of circulating and emerging respiratory viruses on public health, healthcare systems, and economies underscores the need for innovative strategies. Understanding viral structure and host interactions is essential for guiding vaccine and therapeutic development. Traditional methods such as Western blot, ELISA, PCR and mass spectrometry, while informative, lack single-particle resolution and sensitivity to discern viral heterogeneity—key for analyzing antigen composition on the viral envelope and viral subpopulation that may influence pathogenicity and immune evasion. Flow virometry (FVM) is a sensitive, multiparametric, high-throughput technique that applies flow cytometry principles to detect, quantify, and characterize intact viral particles at single-particle level. By leveraging light scattering and fluorescence, FVM enables the analysis of viral biophysical properties, such as the size and protein abundance of both viral and host-derived antigens on the viral envelope [1].

Notably, the application of FVM single-particle analysis for viral characterization provides valuable insights into viral heterogeneity and facilitates the identification of subpopulations with potentially enhanced infectivity [1,2,3,4,5,6,7,8,9,10,11,12,13,14,15,16,17,18]. Several studies have suggested that host-derived and viral proteins, including glycoproteins, may be differentially incorporated depending on particle size, with these variations correlating to distinct viral fitness and infectivity profiles [7,8,12,13,16,17,18,19]. However, a key challenge in characterizing viral populations is their small size, which complicates the ability to effectively discern them from cellular contaminants or instrument background noise, necessitating the development of more sensitive and specific analytical techniques [2,3]. The fluorescent labeling of viral structural components, such as envelope glycoproteins, combined with advancements in dyes and diagnostic modalities, enables the detection and characterization of low-abundance surface antigens on viruses [1,2,3].

Nanobodies (V_H_Hs) are recombinant antigen-binding variable domains derived from the heavy-chain-only antibodies of *Camelidae* [20]. Their small size (~15 kDa), approximately an order of magnitude smaller than IgGs, and single-domain structure confer key advantages over traditional monoclonal antibodies (mAbs) [21,22,23]. These include high intrinsic affinity, thermodynamic stability, and scalable production across various expression systems [22,23,24,25,26]. Nanobodies have been explored as therapeutics in oncology and as antiviral agents, including candidates such as VHH-72/XVR011, which completed Phase 1 clinical trials for COVID-19 therapy early in the pandemic [27,28,29,30]. Their small size improves epitope access in geometrically restricted antigenic sites, enhancing their diagnostic efficacy over mAbs. Notably, the simple genetic structure of nanobodies permits the design and assembly of multimeric formats, such as fusion to the Fc portion of immunoglobulin G (IgG) (V_H_H-Fc), enhancing binding specificity and avidity [22,24,25,26,31]. We previously characterized a diverse collection of anti-SARS-CoV-2 spike glycoprotein (S) nanobodies developed for antiviral therapeutic applications against SARS-CoV-2 [24]. Both monovalent V_H_H and bivalent V_H_H-Fc nanobody formats exhibited high thermal stability, strong affinity, and broad domain/subunit specificity for SARS-CoV-2 S, demonstrating potent neutralization efficacy in vitro and in vivo [24].

In this study, we evaluated a subset of these nanobodies that target distinct regions of the SARS-CoV-2 S, including the receptor-binding domain (RBD), N-terminal domain (NTD), and subunit 2 (S2) regions. By fluorescently conjugating these nanobodies to FITC, we demonstrate efficient detection and selective labeling of pseudotyped viruses expressing the SARS-CoV-2 S. RBD-targeting nanobodies exhibited the most effective staining, followed by NTD-targeting nanobodies, while S2-specific nanobodies showed limited resolution. Leveraging the engineering flexibility of nanobodies, we developed monovalent V_H_H constructs as well as monovalent and bivalent V_H_H-Fc formats. Evaluation of their labeling efficiency and signal resolution demonstrated that bivalent V_H_H-Fc nanobodies significantly enhanced viral detection, providing a superior resolution of viral particles from background noise compared to monovalent V_H_H or V_H_H-Fc nanobody constructs. This versatile nanobody platform, initially developed for antiviral therapeutic use, demonstrates strong potential for diagnostic application, such as FVM assays, offering a rapid and efficient method to detect and characterize SARS-CoV-2 in biological samples.

## 2. Materials and Methods

### 2.1. FITC Conjugation to Nanobodies

**Figure 1:**
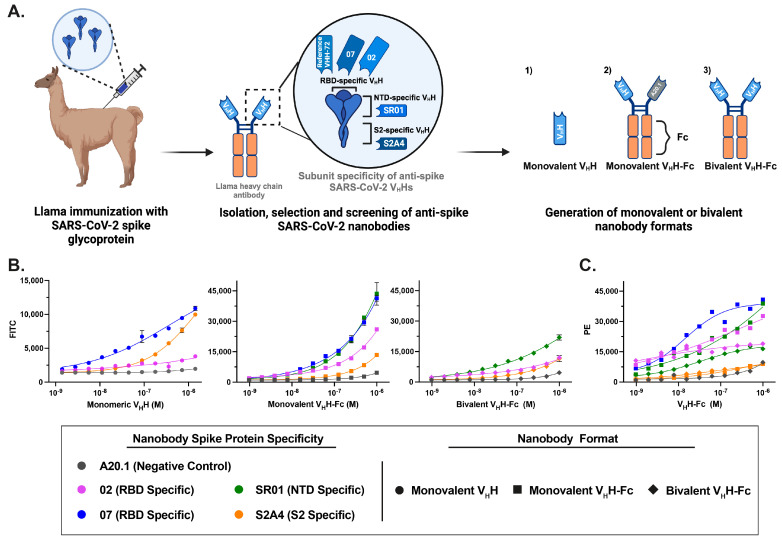
Generation and characterization of fluorescent anti-SARS-CoV-2 spike glycoprotein nanobodies in cell binding assays. (**A**) Schematic representation of the workflow for generating anti-SARS-CoV-2 spike (S) nanobodies targeting the receptor-binding domain (RBD), N-terminal domain (NTD), and S2 subunit. These nanobodies were produced in monovalent V_H_H, monovalent V_H_H-Fc, and bivalent V_H_H-Fc formats. (**B**) Flow cytometry analysis of FITC-conjugated nanobody constructs confirmed their retained binding affinity and specific labeling of CHO cells stably expressing the SARS-CoV-2 spike protein. The values represent the geometric mean of two experiments, with error bars indicating the standard error of the mean (SEM). (**C**) The efficiency of FITC conjugation and the specificity of monovalent and bivalent V_H_H-Fc FITC nanobody constructs were validated by staining with a PE-labeled secondary antibody against the IgG Fc fusion. The values represent the geometric mean of a single experiment.

The V_H_Hs used in this study were previously isolated and characterized [24]. The selected antibodies V_H_H 02, V_H_H 07, V_H_H S2A4, and benchmark VHH-72 [27] were expressed as monomers in bacteria and purified by immobilized metal-ion affinity chromatography (IMAC). Bivalent V_H_H-human IgG1 Fc fusions (02/02; 07/07, SR01/SR01, S2A4/S2A4, and VHH72/VHH72) were obtained by transient transfection of HEK293-6E cells and purified from the supernatant by protein A affinity chromatography. Monovalent V_H_H-Fc molecules were generated by co-transfection with two plasmids: (i) encoding *Clostridioides difficile* toxin A (A20.1)-Fc, C-terminally tagged with a 6×His, and (ii) encoding an untagged V_H_H-Fc specific for SARS-CoV-2 spike protein. The resulting monovalent V_H_H-Fc is a bispecific heterodimer, with one V_H_H targeting the SARS-CoV-2 spike protein and second V_H_H specific for A20.1, an irrelevant nanobody also used in negative controls to assess nonspecific binding [32] (Figure 1A). The heterodimeric bispecific protein was purified by sequential protein A affinity chromatography and IMAC and eluted using a linear 0 to 0.5 M imidazole gradient over 7 column volumes to separate species bearing one (heterodimeric bispecific V_H_H-Fc) or two (A20.1 bivalent V_H_H-Fc) 6×His tags. V_H_Hs and V_H_H-Fcs were buffer-exchanged into phosphate-buffered saline (PBS), pH 7.4. FITC conjugation (Thermo Scientific, Waltham, MA, USA, cat# 46410) was performed following manufacturer’s instructions with minor modifications. A total of 1 mg of each protein was buffer-exchanged into 50 mM borate, pH 8.5, and the labeling reaction was performed at a 1:1 molar ratio to achieve a gentle conjugation level. Free FITC was removed using Amicon^®^ Ultra-15 Centrifugal Filter Units (Millipore-Sigma, St-Louis, MI, USA, cat#UFC905024) by buffer exchange into PBS, pH 7.4. Nanobody constructs were fluorescently conjugated to FITC via lysine residues, either within the V_H_H scaffold for monovalent V_H_Hs or within the Fc region for monovalent and bivalent V_H_H-Fc constructs (Supplementary Table S1). While labeling is more likely to occur within the Fc region than the V_H_H, it remains possible that some labeling occurs within the V_H_H domain in V_H_H-Fc constructs.

### 2.2. Cell Culture

CHO55E1™ cells expressing full-length SARS-CoV-2 Wuhan spike protein (CHO-SPK, NRC, Montreal, Canada) [33] were cultured in BalanCD™ CHO Growth A medium (Irvine Scientific, Santa Ana, CA, USA) supplemented with 50 µM methionine sulfoximine and maintained at 37 °C, 5% CO_2_, and 120 rpm. Expression of the spike protein was induced by adding 2 µg/mL cumate for 48 h at 32 °C.

Human embryonic kidney 293T (HEK-293T) cells (ATCC, CRL-11268) were maintained in Dulbecco’s Modified Eagle Medium (DMEM) (Wisent, Saint-Jean-Baptiste, QC, Canada, cat# 319-005-CL) supplemented with 10% fetal bovine serum (Thermo Fisher Scientific, Waltham, MA, USA,, cat# 12483020) and 100 U/mL penicillin and 100 µg/mL streptomycin (Millipore-Sigma, St-Louis, MI, USA, cat# SV30010).

### 2.3. Flow Cytometry

For flow cytometry of unfixed cells, CHO-SPK cells were harvested by centrifugation, resuspended at 1 × 10^6^ cells/mL in PBS-B (PBS with 1% [*w*/*v*] bovine serum albumin), and kept on ice until use. FITC-labeled V_H_Hs and V_H_H-Fcs were serially diluted 3-fold in PBS-B and mixed with 50 µL of CHO-SPK cells in V-bottom 96-well microtiter plates (Globe Scientific, Mahwah, NJ, USA, Cat# 120130). After 1 h incubation on ice, cells were washed twice with PBS-B (centrifuged for 5 min at 1200 rpm) and resuspended in 50 µL of PBS-B. The binding of FITC-labeled V_H_Hs and V_H_H-Fcs to CHO-SPK cells was assessed using a Beckman Coulter CytoFlex S (Beckman Coulter, Brea, CA, USA) flow cytometer. Data were analyzed with FlowJo™ software (FlowJo LLC, v10.6.2). The same procedure was applied to CHO-SPK cells that had been fixed with 4% paraformaldehyde (*v*/*v*) to assess the recognition by the nanobodies under these conditions. As a positive control and reference for the assay, binding of both unlabeled and FITC-labeled V_H_H-Fcs to the cells was indirectly detected using anti-human IgG Fc. Briefly, antibodies were titrated as described above, cells were washed with PBS-B by centrifugation and then incubated for an additional hour with 50 µL of 250 ng/mL R-Phycoerythrin (R-PE) AffiniPure F(ab’)_2_ Fragment Goat Anti-Human IgG (Jackson ImmunoResearch, Baltimore, PA, USA, cat# 109-116-170) diluted in PBS-B. Following a final wash, cells were resuspended in 50 µL of PBS-B and analyzed as described before.

### 2.4. Plasmids and Pseudotyped Lentivirus Production

Plasmids encoding the SARS-CoV-2 spike glycoprotein and vesicular stomatitis virus glycoprotein (VSV-G) were previously described in Rocheleau et al. [34]. Briefly, a SARS-CoV-2 spike protein variant with a 20-amino-acid C-terminal deletion was generated using overlap extension PCR to introduce a termination codon at residue 1254. HEK-293T cells were transiently co-transfected with the lentiviral packaging plasmid psPAX2 (Addgene, cat# 12260) and plasmids encoding either SARS-CoV-2 spike or VSV-G at a 1:1:1 ratio using GeneJuice transfection reagent (Sigma Aldrich, St-Louis, MI, USA, cat# 70967). Supernatants were harvested 72 h post-transfection and filtered through a 0.45 µm filter.

### 2.5. Western Blot Analysis

HEK-293T cells transfected with SARS-CoV-2 spike or VSV-G were collected 72 h post-transfection, washed with cold PBS, and lysed in 4× Laemmli buffer. Cell supernatants were separated on 4–12% gradient SDS-polyacrylamide gels (NuPage, Invitrogen, Waltham, MA, USA) and transferred to polyvinylidene difluoride (PVDF) membranes. Membranes were blocked for 1 h at room temperature in 5% (*w*/*v*) skim milk dissolved in TBST (25 mM Tris, pH 7.5, 150 mM NaCl, 0.1% (*v*/*v*) Tween 20). Spike protein expression was analyzed by immunoblotting using an anti-S1 polyclonal antibody (1:2000) (Invitrogen, Waltham, MA, USA, cat# PA5-81795) or an anti-S2 monoclonal antibody (1:2000) (Invitrogen, Waltham, MA, USA, cat# MA5-35946). Cell supernatants from the same experiments were processed on separate blots and probed with a goat anti-rabbit horseradish peroxidase (HRP)-conjugated secondary antibody (1:10,000). VSV-G protein expression was confirmed by immunoblotting with an anti-VSV-G polyclonal antibody (1:2000) (GenScript, Pisacataway, NJ, USA, cat# A00199-40), followed by a goat anti-rabbit HRP-conjugated secondary antibody (1:10,000). β-tubulin expression was assessed by immunoblotting cell lysates with an HRP-conjugated anti-β-tubulin antibody (Abcam, Cambridge, UK, cat# ab173840).

### 2.6. Flow Virometry

A comprehensive methodology detailing antibody staining optimization, instrument settings, and calibration is described in Maltseva et al. [2,3]. Briefly, flow virometry (FVM) analysis was performed using a CytoFLEX S (Beckman Coulter, Brea, CA, USA), with 405 nm SSC-H as the threshold parameter (threshold: 1500 a.u.), at a 10 µL/min rate for 60 s. All pseudotyped virus-containing supernatants were filtered, centrifuged, and diluted in 0.1-µm filtered (Pall Corporation, New York, NY, USA, cat# 4611) 1 × PBS (Wisent, Saint-Jean-Baptiste, QC, Canada, cat# 311-430-CL) before staining. Prior to viral staining, antibodies were centrifuged at 17,000× *g* for 10 min to reduce antibody aggregates. Table 1 and Supplementary Table S1 provide a detailed list of FITC-conjugated antibodies used in this study. Viral antibody labeling was performed using a 1:1 ratio of viral supernatant to FITC-conjugated antibody, with a final antibody concentration of 3.2 µg/mL per 1 × 10^9^ viral particles, incubated for 60 min at 37 °C. Stained viral supernatants were diluted in 0.1 µm filtered PBS and acquired for 60 s at a low flow rate of 10 µL/min. Pre-mixed Quantum FITC-5 MESF Beads (Bangs Labs, Fishers, IN, USA, cat# 555) were run in parallel and used for fluorescence calibration to report standardized fluorescence units in FITC molecules of equivalent soluble fluorophores (MESF). Data calibration was performed using FCM_PASS_ (version 3.10.0, https://www.fcmpass.com, URL accessed on 11 September 2022) [35,36,37] with detailed parameters provided in the FCM_PASS_ report (Supplementary Figure S2) and MIFlowCyt-EV checklist (Supporting Information File). Post-calibration analysis was conducted in FlowJo v10.7.1. Supplementary Figure S3 illustrates the gating strategy used for antigen detection, and the median FITC MESF values were quantified from the designated viral gates. Statistical analyses were performed using one-way ANOVA with Tukey’s multiple comparisons in GraphPad Prism (version 9.5.1, GraphPad Software).

Stain index was calculated as follows:

$$\mathrm{Stain}\;\mathrm{index}=\frac{Median\;MESF\;of\;labeled\;\;population-Median\;MESF\;of\;VSV-G\;labeled\;\;control\;}{SD\;of\;VSV-G\;labeled\;control}$$

## 3. Results

### 3.1. Generation of Fluorescent Anti-SARS-CoV-2 Spike Glycoprotein Nanobodies

Previously, we conducted a comprehensive characterization of a diverse panel of anti-SARS-CoV-2 spike glycoprotein nanobodies developed for antiviral therapeutic applications [24]. To generate these nanobodies, a llama was immunized with the Wuhan Hu-1 (Wu-1) spike glycoprotein and boosted with three doses of the receptor-binding domain (RBD), eliciting a robust humoral response against the full-length spike and its S1 and S2 subunits, yielding 37 unique V_H_Hs targeting distinct epitopes (Figure 1A). These V_H_Hs were engineered into three distinct formats: (i) monovalent V_H_Hs, generated by cloning the sequences as fusions to a biotinylation acceptor peptide (BAP) and a 6×His tag, followed by expression in *Escherichia coli*, and (ii/iii) Fc-fusion V_H_Hs (V_H_H-Fcs), produced by cloning the sequences as fusions with the human IgG1 Fc region to generate either monovalent or bivalent nanobody constructs, as previously described [32]. The resulting monovalent V_h_H-Fc is a bispecific heterodimer, with one V_H_H targeting the SARS-CoV-2 spike protein and second V_H_H specific for A20.1, an irrelevant nanobody also used in negative controls to assess nonspecific binding [38] (Figure 1A). The V_H_H-Fcs were subsequently expressed in HEK293-6E cells. The generated monovalent V_H_Hs and bivalent V_H_H-Fc formats were further characterized by surface plasmon resonance (SPR), flow cytometry and ELISA against recombinant Wu-1 SARS-CoV-2 S, RBD, S1, NTD, and S2 proteins to determine their affinities and subunit/domain specificities as published in Rossotti et al. [24]. These nanobodies exhibited high-affinity binding, with most equilibrium dissociation constants (*K*_D_s) in the single-digit nanomolar to picomolar range (Table 1). Three major specificity clusters were identified: RBD-specific, NTD-specific, and S2-specific V_H_Hs. For this study, we selected two RBD-specific (02, 07), one NTD-specific (SR01), and one S2-specific (S2A4) nanobody as potential diagnostic tools for FVM (Figure 1A).

To assess non-specific labeling, a negative control nanobody targeting *C. difficile* toxin A (A20.1) was generated in both V_H_H and V_H_H-Fc formats [32]. The evaluated nanobody constructs were fluorescently conjugated to FITC via lysine residues, either within the V_H_H scaffold for monovalent V_H_Hs or within the Fc region for monovalent and bivalent V_H_H-Fc constructs (Supplementary Table S1). While labeling is more likely to occur within the Fc region than the V_H_H of monovalent or bivalent V_H_H-Fc constructs, it remains possible that some labeling may occur within the V_H_H domain. The SR01 nanobody was evaluated only in monovalent and bivalent V_H_H-Fc formats due to its low production yield as a monovalent V_H_H in *E. coli*. VHH-72, a previously characterized SARS-CoV S-specific nanobody that cross-reacts with SARS-CoV-2 S, served as a benchmark control [27].

### 3.2. Characterization of Fluorescent Anti-SARS-CoV-2 Spike Glycoprotein Nanobodies for Cell Binding Assays

The developed fluorescent conjugated nanobodies in the three formats (monovalent V_H_H, monovalent V_H_H-Fc, and bivalent V_H_H-Fc) were evaluated for diagnostic application. Flow cytometry assays confirmed that FITC-conjugated nanobodies retained binding affinity and specifically recognized CHO cells stably transfected with the SARS-CoV-2 S protein (CHO-SPK) (Figure 1B). Due to reagent limitations, the bivalent V_H_H-Fc 07 FITC-conjugated construct was not included in the cell-binding assays; however, its specificity and binding affinity were previously characterized by Rossotti et al. [24]. RBD- and NTD-targeting nanobodies exhibited the strongest staining, while S2-specific nanobodies showed reduced resolution at lower concentrations (Figure 1B).Compared to monovalent and bivalent V_H_H-Fc formats, monovalent V_H_Hs exhibited reduced labeling resolution across the tested concentration range. This suggests that FITC conjugation to lysine residues within the V_H_H, particularly in the complementarity-determining regions (CDRs) as defined by the IMGT system—which are directly involved in antigen interaction (Supplementary Table S1)—may interfere with binding, as previously reported [39,40,41]. This effect was evident in the significant loss of binding observed for the RBD-specific V_H_H 02 and the reduced binding of the V_H_H S2A4 relative to the V_H_H 07. This effect was not observed in bivalent V_H_H-Fc antibodies, suggesting that FITC conjugation to lysine residues in the Fc region does not disrupt antigen binding, likely due to its distance from the binding domains. Additionally, the Fc domain provided a larger surface area for the conjugation of FITC molecules, thereby increasing the sensitivity of the assay. The efficacy of FITC conjugation and the specificity of monovalent and bivalent V_H_H-Fc FITC nanobody constructs for the SARS-CoV-2 S protein were validated by staining against the IgG Fc fusion using a PE-labeled secondary antibody (Figure 1C).

Since FVM can be used with both pseudotyped and live SARS-CoV-2, the latter requiring biosafety level (BSL) 3 containment, next, we sought to confirm whether these nanobody constructs retain their binding specificity following commonly used fixation methods. To this end, we first fixed S-expressing CHO cells with 4% paraformaldehyde and then stained them with FITC-conjugated nanobodies. The staining patterns and resolution were comparable to unfixed samples, indicating suitability for fixation-dependent applications (Supplementary Figure S1). Overall, we demonstrate effective staining of S-expressing CHO cells with minimal non-specific binding, confirming that all three FITC-conjugated nanobody formats are suitable for diagnostic applications requiring direct fluorescence labeling under both BSL-2 or BSL-3 conditions.

### 3.3. Characterization of Fluorescent Anti-SARS-CoV-2 Spike Glycoprotein Nanobodies for Flow Virometry Applications

**Figure 2:**
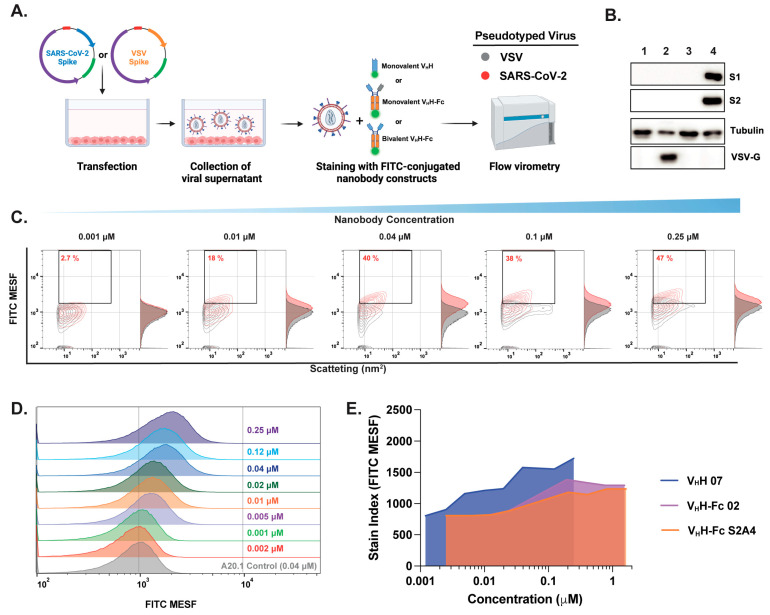
Optimization of fluorescent anti-SARS-CoV-2 spike nanobody staining for effective SARS-CoV-2 labeling and resolution in flow virometry applications. (**A**) Schematic representation of pseudotyped virus generation and staining with FITC-conjugated nanobodies for downstream FVM analysis. Pseudotyped viruses expressing the SARS-CoV-2 spike protein on their envelope (red) were compared to viruses expressing the vesicular stomatitis virus spike glycoprotein (VSV-G) (gray), which served as a negative control to assess non-specific binding. (**B**) Confirmation of S (S1 and S2 subunits) and VSV-G protein expression in viral supernatants via Western blot analysis. (**C**) Contour plots and (**D**) histogram plots showing the titration of RBD-specific monovalent V_H_H 07 over a concentration range of 0.002–0.25 µM. (**E**) Stain index comparison for monovalent V_H_H 07, bivalent V_H_H-Fc 02, and S2A4 nanobody constructs for the determination of the optimal staining concentration that provides minimal nonspecific binding. The stain index was calculated using the median fluorescent intensity in FITC MESF of positively and negatively labeled virus populations divided by the standard deviation of the negative population. The values represent the geometric mean of a single experiment. MESF, molecule of equivalent soluble fluorochrome.

We previously established a workflow for FVM instrument setup and calibration using reference materials to optimize single-particle sensitivity [3]. Fluorescence signals were quantified in FITC-MESF units using calibration beads and FCM_PASS_ software, which standardizes light scatter and fluorescence measurements with commercial reference standards, ensuring compliance with the MIFlow-Cyt-EV framework (Supplementary Figure S2 and Supporting Information File) [35,36,37]. This enabled the accurate detection and quantification of viral particle concentration and S protein abundance following labeling with fluorescently conjugated nanobodies. To this end, we generated two pseudotyped viruses: one expressing the SARS-CoV-2 S protein on the viral envelope and another expressing the vesicular stomatitis virus glycoprotein (VSV-G). The latter served as an additional negative control to assess non-specific binding (Figure 2A). The expression of SARS-CoV-2 S or VSV-G on pseudotyped viruses was assessed by the immunoblotting of viral supernatants, probing for the S1 and S2 subunits of SARS-CoV-2 S or the VSV-G protein, respectively (Figure 2B).

Next, we selected three nanobodies (monovalent V_H_H 07, bivalent V_H_H-Fc 02, or S2A4) and performed staining titrations to quantify S protein expression using optimized techniques and appropriate controls, including PBS-only, media with nanobody, and VSV-G or S pseudotyped virus with nanobody. SARS-CoV-2 S pseudotyped viruses were resolved by light scatter and fluorescence using the gating strategy depicted in Supplementary Figure S3. Optimizing antibody staining via titration is crucial for resolving dimly stained virus particles from unstained populations and background noise. Excess antibody concentrations increase background fluorescence and non-specific binding, as shown in Figure 2C. A key challenge in FVM is the limited dynamic range for detecting and resolving stained viral particles, which is strongly influenced by antigen expression levels on the viral envelope and background fluorescence. This limitation can impair the resolution of viral particles with low antigen expression. Although only ~40% of viral particles were FITC-positive within the 0.04–0.25 µM range, contour and histogram plots show a clear shift in FITC signal and improved resolution of the majority of viral particles, particularly at 0.04 µM, relative to the negative control and VSV-G-pseudotyped controls (Figure 2C,D). To achieve optimal separation between positively and negatively labeled populations, the stain index was calculated (Figure 2E). A concentration of 3.4 µg/mL (0.04 µM for V_H_H or 0.2 µM for V_H_H-Fc) was selected for all subsequent staining with the full panel of monovalent V_H_H or monovalent and bivalent V_H_H-Fc nanobody formats.

### 3.4. Nanobody Labeling Enables Enhanced Detection and Resolution of SARS-CoV-2 in Flow Virometry

**Figure 3:**
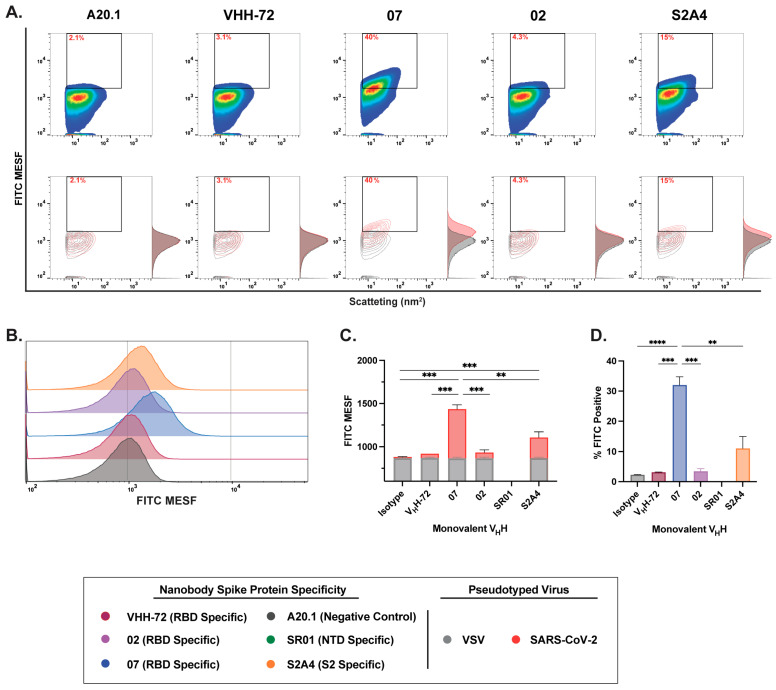
Characterization of binding dynamics and resolution efficiency of monovalent VHH fluorescent staining of SARS-CoV-2 viral particles by FVM. (**A**) Characterization of anti-SARS-CoV-2 spike monovalent V_H_Hs targeting the receptor-binding domain (RBD), N-terminal domain (NTD), and S2 subunit. Dot plots show staining profiles overlaid with VSV-G pseudotyped virus as a negative control (contour plots). (**B**) Histogram plots of monovalent V_H_H staining, represented as FITC MESF signal relative to a VSV-G control. (**C**) Bar graphs displaying FITC MESF signals and (**D**) percentage of FITC-positive viral particles relative to VSV-G and A20.1 negative controls, as determined by FVM using the gating strategy described in Supplementary Figure S3A. Data represent the average of two independent experiments. Statistical analysis was performed by a one-way ANOVA, followed by Tukey’s multiple comparisons test. **** *p* ≤ 0.0001, *** *p* ≤ 0.001, ** *p* ≤ 0.002. MESF, molecule of equivalent soluble fluorochrome.

After validating our experimental setup for effectively detecting and resolving SARS-CoV-2 pseudotyped viral particles relative to the negative VSV-G control, we characterized the binding dynamics and resolution efficiency of RBD-, NTD-, and S2-specific nanobodies in monovalent V_H_H, as well as monovalent and bivalent V_H_H-Fc formats. Consistent with our cell binding flow cytometry analysis, we show that staining with RBD-specific 07 and S2-specific S2A4 monovalent V_H_Hs resulted in detectable viral labeling and resolution relative to the negative or isotype control, as visualized in contour and histogram plots (Figure 3A,B).

Consistent with our cell binding flow cytometry analysis, we show that staining with RBD-specific 07 and S2-specific S2A4 monovalent V_H_Hs resulted in detectable viral labeling and resolution relative to the negative or isotype control, as visualized in contour and histogram plots (Figure 3A,B). Labeling with V_H_H 07 generated the strongest staining, with a significantly higher FITC signal and the highest percentage of FITC-positive particles detected (Figure 3C,D). In contrast, V_H_H S2A4 exhibited moderate staining, with a FITC signal significantly higher than the A20.1 negative nanobody control but lower than that of V_H_H 07. Staining with the benchmark control VHH-72, a previously characterized SARS-CoV S-specific nanobody that cross-reacts with SARS-CoV-2 S [27], did not result in detectable viral resolution (Figure 3C,D). As observed in cellular analysis, staining with the RBD-specific V_H_H 02 did not effectively resolve viral particles from the background. This suggests that FITC conjugation to lysine residues within the CDR

3 region of V_H_H 02 or V_H_H S2A4, as shown in Supplementary Table S1, may have disrupted antigen recognition or significantly reduced binding affinity to S protein (Figure 3C,D). As we previously demonstrated in Rossotti et al. [24], unlabeled V_H_H 02 exhibited strong binding to S-expressing CHO cells, supporting the conclusion that FITC conjugation impaired antigen binding and led to reduced labeling.

**Figure 4:**
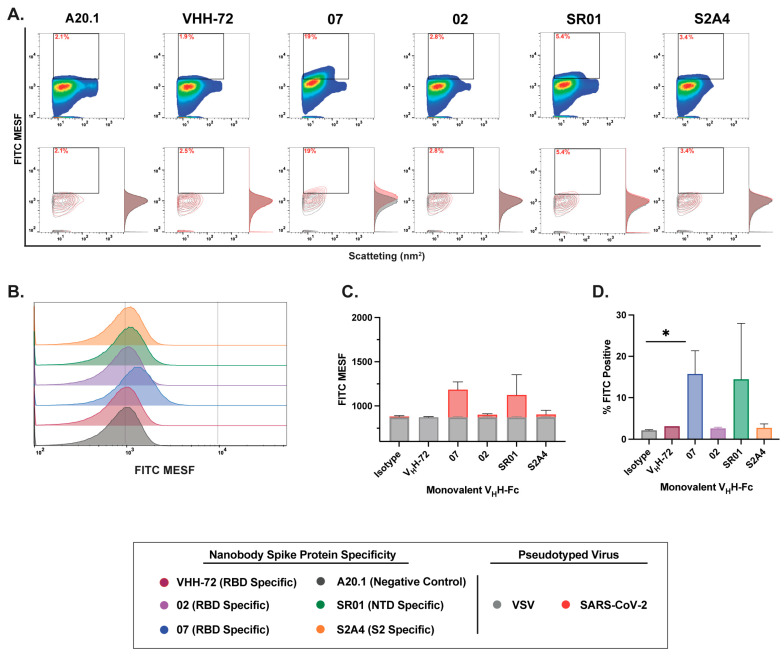
Characterization of binding dynamics and resolution efficiency of monovalent V_H_H-Fc fluorescent staining of SARS-CoV-2 viral particles by FVM. (**A**) Characterization of anti-SARS-CoV-2 spike monovalent V_H_H-Fcs targeting the receptor-binding domain (RBD), N-terminal domain (NTD), and S2 subunit. Dot plots show staining profiles overlaid with VSV-G pseudotyped virus as a negative control (contour plots). (**B**) Histogram plots of monovalent V_H_H-Fcs staining, represented as FITC MESF signal relative to VSV-G control. (**C**) Bar graphs displaying FITC MESF signals and (**D**) percentage of FITC-positive viral particles relative to VSV-G and A20.1 negative controls, as determined by FVM using the gating strategy described in Supplementary Figure S3A. Data represent the average of two independent experiments. Statistical analysis was performed by a one-way ANOVA, followed by Tukey’s multiple comparisons test. * *p* ≤ 0.05. MESF, molecule of equivalent soluble fluorochrome.

Next, we evaluated the binding dynamics and resolution efficiency of RBD-, NTD-, and S2-specific nanobodies in monovalent and bivalent V_H_H-Fc formats. In contrast to the binding dynamics observed with monovalent V_H_H and cellular binding assessments, monovalent V_H_H-Fc nanobody constructs exhibited moderate to low binding efficiency and reduced resolution of SARS-CoV-2 S-pseudotyped viral particles (Figure 4A,B). While staining with RBD-specific V_H_H 07 and NTD-specific V_H_H SR01 monovalent V_H_H-Fc nanobodies showed a positive trend in FITC intensity and the resolution of FITC-positive particles relative to the A20.1 negative isotype or VSV-G controls (Figure 4), this increase was not statistically significant. However, staining with the RBD-specific V_H_H 07 monovalent V_H_H-Fc resulted in a significantly higher number of labeled viral particles (Figure 4D).

**Figure 5:**
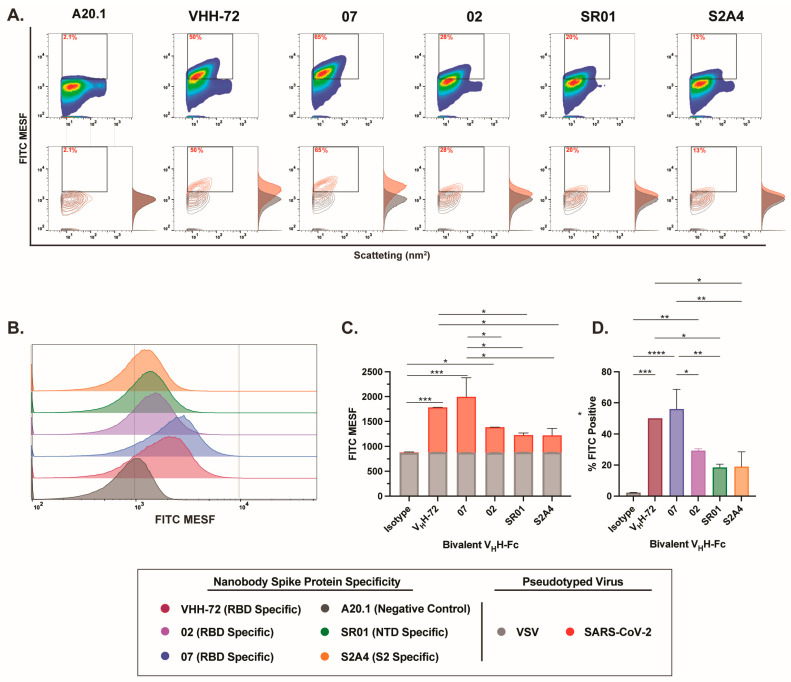
Characterization of binding dynamics and resolution efficiency of bivalent V_H_H-Fc fluorescent labeling of SARS-CoV-2 viral particles by FVM. (**A**) Characterization of anti-SARS-CoV-2 spike bivalent V_H_Hs targeting the receptor-binding domain (RBD), N-terminal domain (NTD), and S2 subunit. Dot plots show staining profiles overlaid with VSV-G pseudotyped virus as a negative control (contour plots). (**B**) Histogram plots of monovalent V_H_H staining, represented as FITC MESF signal relative to VSV-G control. (**C**) Bar graphs displaying FITC MESF signals and (**D**) percentage of FITC positive viral particles relative to VSV-G and A20.1 negative controls, as determined by FVM using the gating strategy described in Supplementary Figure S3A. Data represent the average of two independent experiments. Statistical analysis was performed by a one-way ANOVA, followed by Tukey’s multiple comparisons test. **** *p* ≤ 0.0001, *** *p* ≤ 0.001, ** *p* ≤ 0.002, * *p* ≤ 0.05. MESF, molecule of equivalent soluble fluorochrome.

Lastly, labeling with bivalent V_H_H-Fc nanobodies resulted in the most effective staining and the highest resolution efficiency among all evaluated nanobody formats (Figure 5A,B). Notably, staining with RBD-specific nanobodies (V_H_H 02, V_H_H 07, and VHH-72) yielded the most effective labeling, with the majority of labeled particles clearly distinguishable from the background, as illustrated in both contour and histogram plots (Figure 5A). Notably, bivalent V_H_H-Fc 07 and VHH-72 demonstrated the greatest separation from the A20.1 negative isotype or VSV-G controls, indicating superior specificity and signal resolution (Figure 5A,B). Of note, while staining with NTD-specific SR01 and S2-specific S2A4 V_H_H-Fc nanobodies showed a positive trend in FITC signal and the resolution of FITC-positive particles relative to the negative controls (Figure 5C,D), this did not lead to statistical significance. Given the trimeric structure of the S protein, our results provide a direct comparison between monovalent and bivalent V_H_H-Fc constructs, highlighting that multimeric binding to the S antigen increases avidity and consequently enhances the staining efficiency of the bivalent V_H_H-Fc constructs. This is evident from the improved staining observed with bivalent V_H_H-Fc 02, S2A4, and VHH-72 nanobodies relative to the monovalent V_H_H-Fc constructs. These findings suggest that bivalent nanobody constructs may be particularly well suited for targeting complex, multidomain protein assemblies, such as viral glycoproteins, and that they may be suitable for FVM applications.

## 4. Discussion

Nanobodies represent a major advancement in biotherapeutics and diagnostics, offering high specificity, robust stability, and scalable production across various expression systems. Their small size enables better epitope access, especially in geometrically restricted antigenic sites, providing a key advantage over mAbs in diagnostic assays [21,22,23]. Additionally, their simple genetic structure allows for multimeric designs, such as Fc fusion, which may improve binding specificity and avidity. In our previous study [24], we characterized a panel of anti-SARS-CoV-2 S nanobodies for antiviral applications. Both monovalent V_H_H and bivalent V_H_H-Fc nanobodies demonstrated high thermal stability, strong affinity, and broad S protein domain/subunit specificity, demonstrating potent in vitro and in vivo neutralization. Here, we directly compared monovalent V_H_H, monovalent V_H_H-Fc, and bivalent V_H_H-Fc nanobody formats for FVM application.

FVM is a rapid, sensitive, high-throughput technique for quantifying viral concentration, size, and antigen abundance on the viral envelope. We demonstrate that direct staining of viral supernatant with bivalent V_H_H-Fc nanobodies enhances viral detection, offering superior particle resolution over monovalent V_H_H and monovalent V_H_H-Fc formats. However, monovalent V_H_H may better access geometrically restricted sites, such as S2, where the Fc region of V_H_H-Fc can cause steric hindrance and impair labeling efficiency. Overall, we highlight the nanobody platform’s versatility and strong potential for diagnostic applications, such as FVM assays, enabling rapid SARS-CoV-2 detection and characterization in biological samples.

Direct antibody staining enhances assay’s specificity, eliminates cross-reactivity from secondary labeling reagents, minimizes background noise in FVM, and streamlines staining protocols. Here, we demonstrate that fluorescent conjugation of nanobodies to FITC enabled efficient labeling and detection of S-expressing cells and SARS-CoV-2 S-pseudotyped viruses. However, certain V_H_H constructs (V_H_H 02 and V_H_H S2A4) exhibited reduced or abolished binding in both cell and viral assays, suggesting that random FITC conjugation to lysine residues interfered with antigen recognition. Notably, several lysine residues were identified in the CDRs, which mediate antigen interactions. Specifically, lysine residues, which were detected in the CDR3 regions of monovalent V_H_H 02 and S2A4, were absent in other evaluated nanobodies, such as V_H_H 07, where fluorescent conjugation to the V_H_H framework regions did not interfere with antigen binding in cell or viral assays. This difference may explain the impaired binding observed with monovalent V_H_H 02 and S2A4. These findings align with previous studies showing that lysine modifications in CDRs can disrupt antigen binding by altering critical interaction sites [39,40,42]. To mitigate this issue, site-specific labeling strategies, such as cysteine-based conjugation or sortase A-mediated tagging at pre-defined positions, can preserve antigen-binding activity while minimizing interference from fluorescent conjugation [39,40,42]. Notably, in bivalent V_H_H-Fc formats, the reduced binding of V_H_Hs 02 and VHH-72 was restored, suggesting that FITC conjugation to lysine residues in the Fc region not only provided a greater surface area for fluorescent labeling but also reduced the modification of lysine residues within the antigen-binding domain, preventing the disruption of antigen binding and minimizing reduced staining. Additionally, this could also be attributed to the Fc region’s distance from the antigen-binding domain, reducing steric hindrance or disruption of epitope interactions. Nanobody Fc fusion formats can facilitate fluorophore conjugation and provide a recognizable region for secondary reagents in immunoassays, enhancing signal amplification when required.

The simple genetic structure of nanobodies allows for the generation of multimeric nanobody formats, enhancing binding specificity and avidity [21,22,23,25,26]. In a direct comparison between monovalent V_H_H, monovalent V_H_H-Fc, and bivalent V_H_H-Fc, we observed that the bivalent V_H_H-Fc format provided the most effective staining, successfully distinguishing viral particles from background noise, in contrast to the monovalent V_H_H and V_H_H-Fc nanobody formats. While monovalent V_H_Hs exhibited reduced labeling resolution, staining with monovalent V_H_H 07 and S2A4 resulted in significantly higher FITC signal intensity and a greater percentage of FITC-positive particles compared to the benchmark VHH-72 and the negative controls. Indeed, S2-specific monovalent V_H_H formats demonstrated improved staining efficiency and signal resolution compared to monovalent V_H_H-Fc. This suggests that the smaller size of the monovalent V_H_H (~15 kDa) be more advantageous for accessing geometrically restricted antigenic sites, such as the S2 subunit of SARS-CoV-2 S protein. However, given that only a single S2-specific nanobody was evaluated in this study, future investigations should assess whether other S2-targeting nanobodies exhibit improved staining when expressed in a monovalent V_H_H relative to the V_H_H-Fc format. In contrast, the larger size of V_H_H-Fc formats (~80 kDa), combined with the presence of the Fc region, may introduce steric hindrance, limiting epitope accessibility and reducing labeling efficiency [22,23,26]. While this study directly compares monovalent V_H_H and both monovalent and bivalent V_H_H-Fc formats to benchmark VHH-72 nanobody, future work should incorporate conventional murine or human IgGs specific to SARS-CoV-2 to more comprehensively assess labeling efficiency and signal resolution of nanobodies in FVM applications.

A key challenge in FVM analysis is the compressed dynamic range when distinguishing between positive and negative signals during viral surface antigen staining [1,2,3,43,44]. This limitation primarily arises from the significantly smaller particle size, reduced surface area, and inherently lower antigen density of the viral envelope, which collectively diminish the staining intensity achievable in FVM. These factors further restrict the dynamic range and impact the efficiency of antigen labeling and signal resolution. Specifically, our previous findings indicate that labeling highly abundant antigens on the cell surface results in a broad dynamic range of approximately 2 to 2.5 logs, whereas viral labeling is typically constrained to a narrower range of 1 to 1.5 logs [2,3]. This reduced dynamic range in viral labeling underscores the need for optimized detection strategies to enhance sensitivity and resolution in FVM applications. Bivalent V_H_H-Fc constructs enhance affinity through simultaneous engagement of multiple antigenic sites, promoting avidity and thereby improving labeling efficiency, particularly for low-abundant antigens [21,22,23,25,26,31]. This avidity-mediated increase in apparent binding affinity enhances detection sensitivity, as evidenced by studies on SARS-CoV-2, where bi- and tri-valent V_H_H-Fc constructs demonstrated improved neutralization potency and antigen recognition [24,45,46]. Thus, staining with bivalent V_H_H-Fc nanobody format may provide a significant advantage in FVM by enhancing both sensitivity and resolution in viral detection.

Although this study specifically evaluates the ability of nanobodies to detect the spike glycoprotein of the ancestral Wu-1 SARS-CoV-2 strain, Rossotti et al. have extensively characterized these nanobodies and demonstrated their binding capacity to various SARS-CoV-2 variants of concern and other animal coronaviruses [24]. Here, we demonstrate that viruses in the supernatants of infected cells can be directly stained and identified using light scatter and fluorescence without the need for additional concentration or manipulation. FVM is a powerful tool for single-particle viral analysis, providing insights beyond traditional bulk methods. Its capacity to resolve viral heterogeneity and sort infectious particles for downstream applications enhances virological research. Furthermore, nanobody-based diagnostics offer a promising approach for FVM, addressing inherent limitations while enhancing antigen labeling efficiency, binding specificity, and detection resolution. These advantages make them well-suited diagnostic tools for characterizing viral pathogens, including SARS-CoV-2, in biological samples.

## 5. Conclusions

Our study highlights the strong potential of nanobody-based diagnostics for enhancing FVM in detecting SARS-CoV-2. We demonstrated that bivalent V_H_H-Fc nanobodies offer superior staining resolution and sensitivity, effectively distinguishing viral particles from background noise. Although monovalent V_H_Hs can access geometrically restricted sites, such as the S2 domain, their performance may be hindered when fused with an Fc region due to steric effects. Moreover, our observations regarding FITC conjugation indicate that random labeling on lysine residues within critical binding domains can compromise antigen recognition, emphasizing the need for refined, site-specific conjugation strategies.

Overall, the inherent advantages of nanobodies—such as their high specificity, robust stability, and versatility in multimeric design—support their utility in rapid, sensitive, and high-throughput diagnostic assays. These findings not only reinforce the potential of nanobody platforms for antiviral applications but also pave the way for further optimization of FVM techniques. Future studies should focus on enhancing labeling methodologies and expanding this approach to a broader array of viral pathogens, ultimately contributing to improved diagnostic capabilities in clinical and research settings.

## Figures and Tables

**Table 1:** Binding characteristics of SARS-CoV-2 nanobodies ^1^.

		Format of Nanobody	
Spike Protein Domain/Subunit	Nanobody Name	Monovalent V_H_H SPR (*K*_D_, nM)	Bivalent V_H_H-Fc Flow Cytometry (*EC*_50_, nM)
RBD	VHH-72 ^2^	86.2	0.2
	07	0.94	0.3
	02	0.62	1
NTD	SR01	0.56	3.4
S2	S2A4	12.8	0.1

^1^ Reported *K*_D_ and *EC*_50_ values correspond to binding measurements for the full trimeric spike protein of the Wuhan SARS-CoV-2 [24]. ^2^ The VHH-72 reference is a SARS-CoV S-specific V_H_H that cross-reacts with SARS-CoV-2 S [27].

## Data Availability

The raw data supporting the conclusions of this article will be made available by the authors on request.

## Supplementary Materials

The following supporting information can be downloaded at https://www.mdpi.com/article/10.3390/v17040571/s1, Supplementary Figure S1: FITC-conjugated nanobody constructs retain binding specificity after fixation of target cells; Supplementary Figure S2: Calibration of fluorescence and light scatter using FCM_PASS_ for standardized data reporting and instrument performance evaluation; Supplementary Figure S3: Gating strategy for phenotypic analysis of SARS-CoV-2 spike glycoproteins on the surface of pseudotyped viruses. Supplementary Table S1: Amino acid sequences of the V_H_Hs and human IgG Fc used for constructing monovalent and bivalent nanobodies. Supporting Information File: FCM_PASS_ software output report.
