# Supplementary material for: Characterization of Nanobody Binding to Distinct Regions of the SARS-CoV-2 Spike Protein by Flow Virometry
Source: Viruses. 2025 Apr 15;17(4):571. doi: 10.3390/v17040571 (PMC12030927; doi:10.3390/v17040571)
Supplement: Supplementary file 1 [file viruses-17-00571-s001.zip › viruses-3527353-supplementary.pdf]

SUPPLEMENTARY INFORMATION

Characterization of Nanobody Binding to Distinct Regions of the SARS-CoV-2 Spike Protein by Flow Virometry

Mariam Maltseva[1], Martin A. Rossotti[2], Jamshid Tanha[1,2,3], and Marc-André Langlois[1,3,*]

[1]Department of Biochemistry, Microbiology and Immunology, Faculty of Medicine, University of Ottawa, Ottawa, ON, Canada.

[2]Human Health Therapeutics Research Centre, Life Sciences Division, National Research Council Canada, Ottawa, ON, Canada

[3]uOttawa Center for Infection, Immunity, and Inflammation (CI3), Ottawa, ON, Canada

*Correspondence should be addressed to: langlois@uottawa.ca

Supplementary Table S1: Amino acid sequences of the V_HHs and human IgG Fc used for constructing monovalent and bivalent nanobodies.

V_HH/Fc	Amino Acid Sequence
A20.1 [1]	QVQLVESGGGLAQAGGSLRLSCAAS**GRTFSMDP**MAWFRQPPGKEREFVAA**GSSTG**<br>**RTT**YYADSVKGRFTISRDNAKNTVYLQMNSLKPEDTAVYYC**AAAPYGANWYRDEY**<br>**AY**WGQGTQVTVSSGQAGQGGGLNDIFEAQKIEWHELEHHHHHH
02	EVQLVESGGGLVQAGGSLRLSCAAS**GFTFSNYA**MNWVRQAPGKGLEWVSG**ISGRG**<br>**DDT**RYADSVKGRFTISRDNAKNTLFLQMRSLRPEDTGVYRC**TKGPDLYYFGSGYSD**<br>RGQGTQVTVSSGQAGQGGGLNDIFEAQKIEWHELEHHHHHH
07	QVQLVQSGGGLVQPGGSLRLSCAAS**GVTLDYYA**IGWFRQAPGKEREAVSC**ISSNGR**<br>**RN**HYVASVRGRFTISRDNAKSTVYLQMNSLKPEDTAVYYC**AAVQDVHGDNYYCTS**<br>**PNEYNV**WGQGTQVTVSSGQAGQGGGLNDIFEAQKIEWHELEHHHHHH
SR01	EVQLVQSGGGSVQAGGSLRLSCVAS**GFTFDNYA**IGWFRQAPGKEREGVSC**ISGNGG**<br>**VT**VHADSVKGRFTISRDNAKNLVYLQMNSLKPEDTAVYYC**AATGIRSTWSVYGCSR**<br>**LAGPYDY**WGQGTQVTVSSGQAGQGGGLNDIFEAQKIEWHELEHHHHHH
S2A4	QVQLVQSGGGLVQAGGSLRLSCAVS **GSPFRSNV** MEWYRQAPGKQRELVAS<br>**ISTGGSR** TYTDSVKGRFTISRDNAKNEAFLQMNSLKPEDTAVYYC<br>**HAAARDSHGIYLLDT**<br>WGQGTQVTVSSGQAGQGGGLNDIFEAQKIEWHELEHHHHHH
VHH-72 [2]	QVQLQESGGGLVQAGGSLRLSCAAS**GRTFSEYA**MGWFRQAPGKEREFVAT**ISWSG**<br>**GST**YYTDSVKGRFTISRDNAKNTVYLQMNSLKPDDTAVYYC**AAAGLGTVVSEWDY**<br>**DYDY**WGQGTQVTVSSGQAGQGGGLNDIFEAQKIEWHELEHHHHHH
Human IgG1 Fc	EPKSCDKTHTCPPCPAPELLGGPSVFLFPPKPKDTLMISRTPEVTCVVVDVSHEDPEVK<br>FNWYVDGVEVHNAKTKPREEQYNSTYRVVSVLTVLHQDWLNGKEYKCKVSNKALP<br>APIEKTISKAKGQPREPQVYTLPPSRDELTKNQVSLTCLVKGFYPSDIAVEWESNGQPE<br>NNYKTTPPVLDSDGSFFLYSKLTVDKSRWQQGNVFSCSVMHEALHNHYTQKSLSLSP<br>G

The CDRs are boldfaced. Spacer, BAP tag and His_6 tag sequences are underlined. Lysin residues, potential targets for fluorescent conjugation, are in red font. The first 15 amino acids (italic and underlined) of the human IgG1 Fc sequence constitute the hinge region. The IMGT system for CDR designation as described in Lefranc et al.[3].

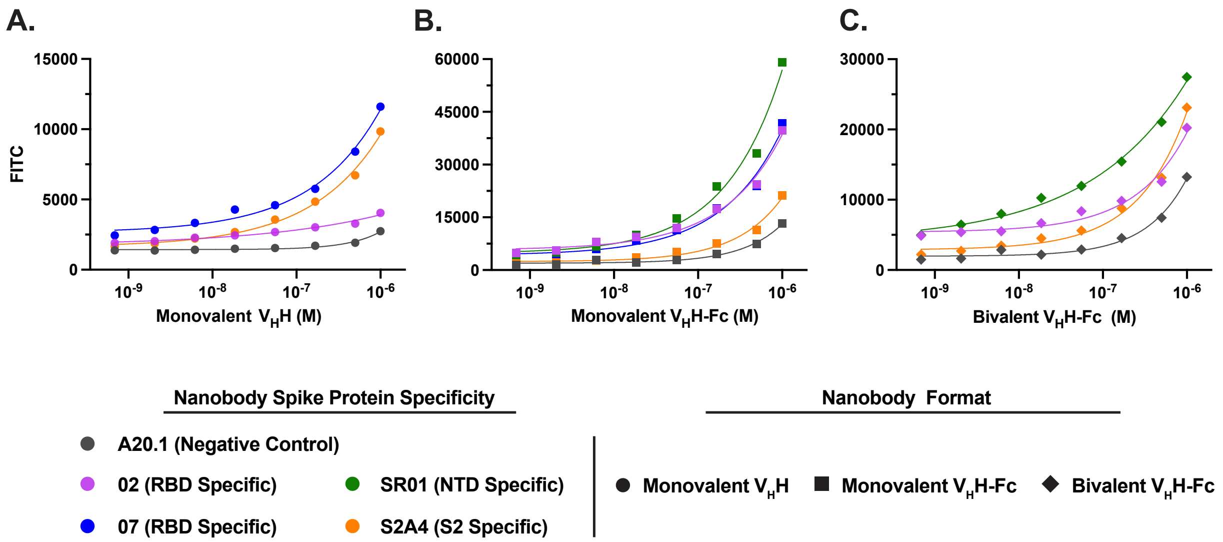

Supplementary Figure S1. FITC-Conjugated Nanobody Constructs Retain Binding Specificity After Fixation of Target Cells. Spike-expressing CHO cells, fixed with 4% paraformaldehyde, were stained with FITC-conjugated nanobodies, displaying staining patterns and resolution comparable to unfixed samples in a dose-dependent manner. **(A)** Monovalent V_HH**,** **(B)** monovalent V_HH-Fc, and **(C)** bivalent V_HH-Fc formats exhibited minimal non-specific binding across the tested nanobody dose range, confirming their suitability for fixation-dependent diagnostic applications. The values represent the geometric mean of one experiment.

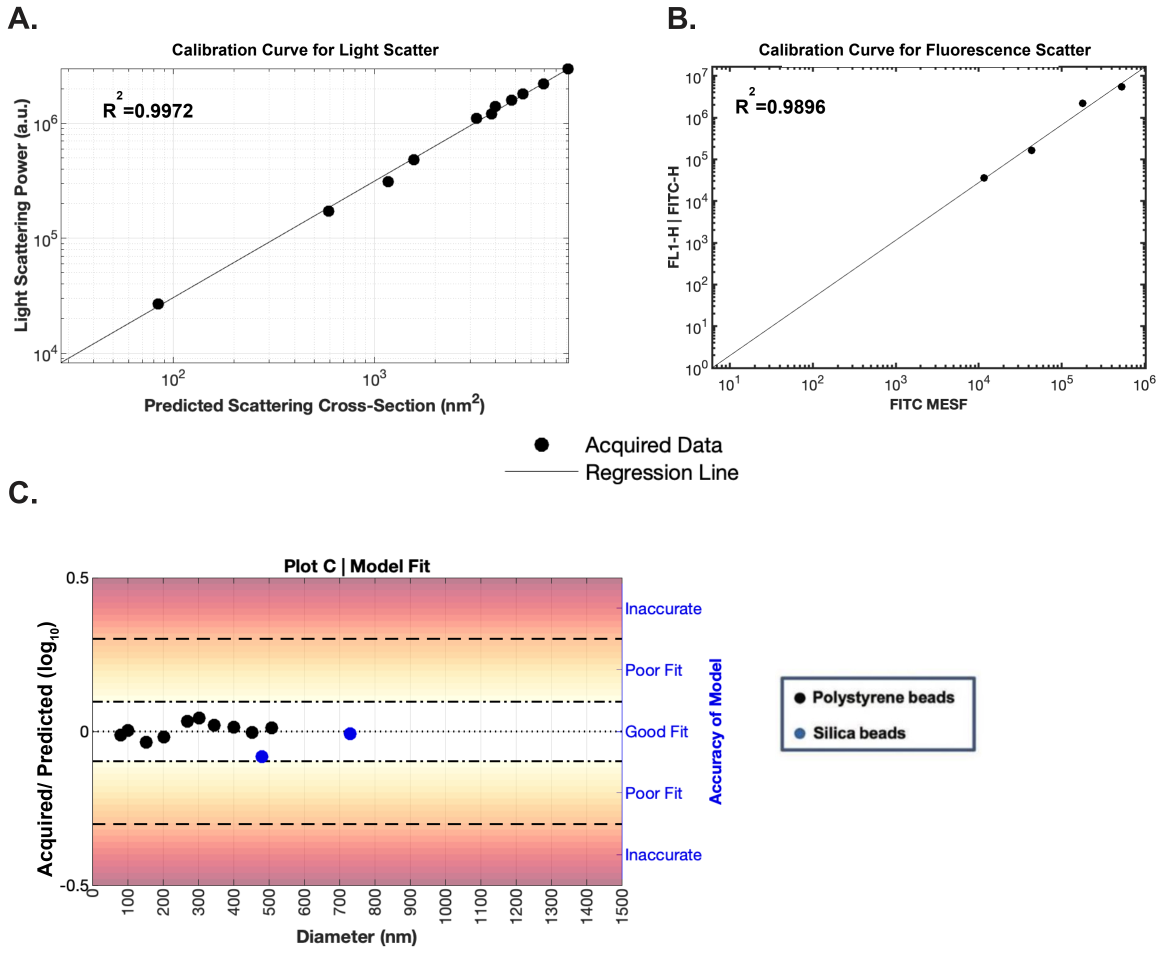

Supplementary Figure S2: Calibration of Fluorescence and Light Scatter Using FCMPASS for Standardized Data Reporting and Instrument Performance Evaluation. Calibration curves for **(A)** light scatter and **(B)** fluorescence outputs generated by FCMPASS software, demonstrating a very strong correlation between the acquired and predicted values of the reference beads. **(C)** Quality control plot validating light scatter standard points fall within the 'good fit' range, as modeled by FCMPASS software.

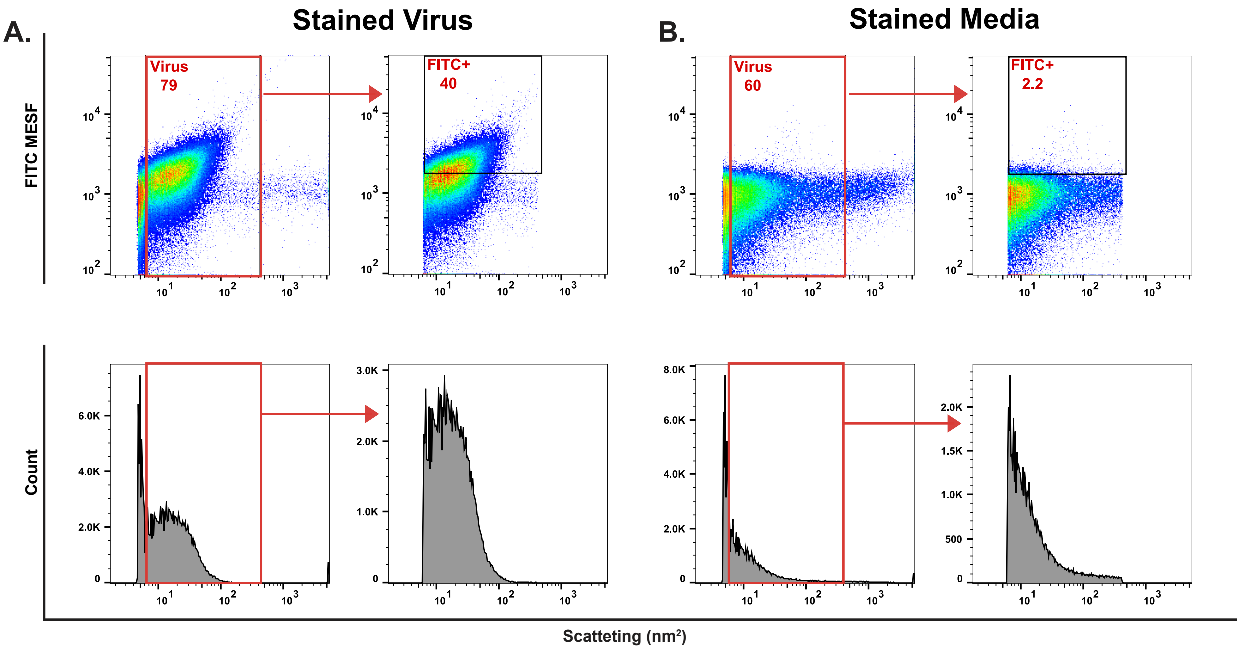

Supplementary Figure S3: Gating Strategy for Phenotypic Analysis of SARS-CoV-2 Spike Glycoproteins on the Surface of Pseudotyped Viruses. **(A)** Stained SARS-CoV-2 spike pseudotyped virus labeled with monovalent V_HH was identified based on FITC fluorescence and side scatter characteristics. **(B)** Stained media-only control was used to assess background fluorescence and antibody aggregates. Background noise within this gate was used to define upper and lower gating thresholds for identifying labeled virions **(top panel).**

Supplementary Information File: FCM_{PASS} software output report. Output report based on the MIFlowCyt-EV report guidelines and summary of the instrument's parameters, the trigger threshold of the particles analyzed, light scatter, and fluorescence calibration. All samples were acquired at identical acquisition settings as the reference beads and were calibrated using FCM_{PASS}.

See attached Excel spreadsheet

References

1. Hussack, G.; Ryan, S.; van Faassen, H.; Rossotti, M.; MacKenzie, C.R.; Tanha, J. Neutralization of Clostridium difficile toxin B with VHH-Fc fusions targeting the delivery and CROPs domains. *PLoS One* **2018**, *13*, e0208978, doi:10.1371/journal.pone.0208978.
2. Wrapp, D.; De Vlieger, D.; Corbett, K.S.; Torres, G.M.; Wang, N.; Van Breedam, W.; Roose, K.; van Schie, L.; Team, V.-C.C.-R.; Hoffmann, M.; et al. Structural Basis for Potent Neutralization of Betacoronaviruses by Single-Domain Camelid Antibodies. *Cell* **2020**, *181*, 1436-1441, doi:10.1016/j.cell.2020.05.047.
3. Lefranc, M.P.; Pommie, C.; Ruiz, M.; Giudicelli, V.; Foulquier, E.; Truong, L.; Thouvenin-Contet, V.; Lefranc, G. IMGT unique numbering for immunoglobulin and T cell receptor variable domains and Ig superfamily V-like domains. *Dev Comp Immunol* **2003**, *27*, 55-77, doi:10.1016/s0145-305x(02)00039-3.
